# Amino acid metabolites that regulate G protein signaling during osmotic stress

**DOI:** 10.1371/journal.pgen.1006829

**Published:** 2017-05-30

**Authors:** James P. Shellhammer, Elizabeth Morin-Kensicki, Jacob P. Matson, Guowei Yin, Daniel G. Isom, Sharon L. Campbell, Robert P. Mohney, Henrik G. Dohlman

**Affiliations:** 1 Department of Pharmacology, University of North Carolina at Chapel Hill, Chapel Hill, North Carolina, United States of America; 2 Metabolon, Inc., Research Triangle Park, North Carolina, United States of America; 3 Department of Biochemistry and Biophysics, University of North Carolina at Chapel Hill, Chapel Hill, North Carolina, United States of America; 4 The University of Miami Miller School of Medicine, Miami, Florida, United States of America; SUNY-Buffalo, UNITED STATES

## Abstract

All cells respond to osmotic stress by implementing molecular signaling events to protect the organism. Failure to properly adapt can lead to pathologies such as hypertension and ischemia-reperfusion injury. Mitogen-activated protein kinases (MAPKs) are activated in response to osmotic stress, as well as by signals acting through G protein-coupled receptors (GPCRs). For proper adaptation, the action of these kinases must be coordinated. To identify second messengers of stress adaptation, we conducted a mass spectrometry-based global metabolomics profiling analysis, quantifying nearly 300 metabolites in the yeast *S*. *cerevisiae*. We show that three branched-chain amino acid (BCAA) metabolites increase in response to osmotic stress and require the MAPK Hog1. Ectopic addition of these BCAA derivatives promotes phosphorylation of the G protein α subunit and dampens G protein-dependent transcription, similar to that seen in response to osmotic stress. Conversely, genetic ablation of Hog1 activity or the BCAA-regulatory enzymes leads to diminished phosphorylation of Gα and increased transcription. Taken together, our results define a new class of candidate second messengers that mediate cross talk between osmotic stress and GPCR signaling pathways.

## Author summary

Just as organisms must adapt to a challenging environment, cells must respond to chemical or physical changes that occur within the organism. Regardless of the environmental cue, all cells use molecular signaling pathways to respond to those changes. Many are transmitted by G protein-coupled receptors (GPCRs) or the high osmolarity glycerol (HOG) pathway. While these pathways have been studied independently for decades, less is known about how they coordinate with each other to carry out the proper response, particularly when conflicting signals are present. One way coordination can be achieved is through “second messenger” molecules produced by one pathway to regulate another pathway. Here, we identify candidate second messengers of osmotic stress by global metabolite profiling analysis of the yeast *S*. *cerevisiae*. We find that three branched-chain amino acid (BCAA) metabolites increase in response to osmotic stress and require the stress response MAPK Hog1. We show that these BCAA derivatives are necessary and sufficient to recapitulate the effects of osmotic stress on the GPCR pathway. Our results identify a new way that HOG and GPCR pathways communicate, and may in the future guide better treatment strategies for stress-related cell damage.

## Introduction

Cells routinely experience changing and often unfavorable conditions in their environment. The ability to adapt to environmental stress and re-establish homeostasis is essential not only to the survival of a cell, but also to the well-being of the organism. The response to such physical or chemical stresses is mediated by well-defined signaling networks. For example, changes in nutrient availability switch signaling between the opposing target of rapamycin (TOR) and AMP-activated protein kinase (AMPK) pathways [1, 2]. Stressors such as UV irradiation, inflammatory cytokines, and osmotic shock promote signaling through activation of the p38 and c-Jun N-terminal Kinase (JNK) MAPK pathways [3, 4]. While much is known about the mechanisms of stress-dependent signaling, less is known about coordination between the stress response and other cell signaling processes. In this study, we investigate cross-talk between osmotic stress and G protein-coupled receptor (GPCR) signaling pathways.

Hyperosmotic stress causes water efflux and cell shrinkage in order to normalize the osmotic balance between the intracellular and extracellular space. Depending on the severity of the stress, cell shrinkage can lead to macromolecular crowding and alterations in cellular protein activity [5], the production of reactive oxygen species (ROS), DNA damage, cell cycle arrest, and apoptosis [6]. In addition to these negative effects, cells also initiate signaling events that promote adaptation. Most prominently, osmotic stress activates the MAPK p38, which in turn phosphorylates myriad downstream targets that coordinate osmotic stress adaptations. Targets of p38 include the transcription factor NFAT5, which promotes the expression of proteins associated with the synthesis and transport of osmolytes, antioxidants, and molecular chaperones [7, 8]. Such changes ensure the survival of the cell, and they are likely to have important consequences for other signaling pathways via cross-talk mechanisms.

As the largest receptor family in humans [9], GPCRs are likely targets of cross-pathway regulation. These receptors respond to a wide variety of homeostatic cues, such as hormones and neurotransmitters, as well as to environmental signals such as odors and light. They signal primarily through intracellular heterotrimeric G proteins, comprised of Gα and Gβγ subunits. G proteins in turn activate downstream effectors leading to the production of second messenger molecules, such as calcium or cAMP, which bind to and activate intracellular protein kinases. Another mechanism of GPCR signaling entails the direct activation of protein kinases upstream of MAPKs [10, 11].

The G protein α subunit is a molecular on/off switch for signaling processes. As such, it is likely to be a critical target for post-translational modifications that regulate GPCR signaling. In fact, several studies have shown that Gα proteins are phosphorylated, resulting in altered affinity for Gβγ subunits or guanine nucleotides [12–20]. In some cases, phosphorylation is the direct result of pathway activation, and thus constitutes a positive or negative feedback. In other cases, phosphorylation is triggered by parallel pathways, and thus constitutes a mechanism of signal coordination or cross-talk. Previously, we showed that Gα is phosphorylated in response to nutrient limitation [21]. Our focus here is Gα regulation by osmotic stress.

Identifying how environmental stress can promote post-translational modification of Gα subunits is necessary to fully understand the mechanisms by which the pathways are coordinated and integrated. Studying the response to environmental stress is often challenging however, due to the expression of multiple protein isoforms and differences in expression among various tissues and cell types. Given these complexities, much can be learned from the analysis of orthologous signaling processes in simpler eukaryotes.

The budding yeast *Saccharomyces cerevisiae* has a stress response pathway and a GPCR signaling pathway with component proteins that are evolutionarily conserved across eukaryotes. The High Osmolarity Glycerol, or HOG, pathway is comprised of a MAPK (Hog1), a MAPK kinase (Pbs2), and MAPK kinase kinases (Ste11, Ssk2/Ssk22). Upon activation, Hog1 phosphorylates cytoplasmic and nuclear proteins that aid in the restoration of osmotic equilibrium through osmolyte synthesis and the induction of stress response genes [22–25]. Hog1 is the yeast ortholog of mammalian p38 [26, 27].

Yeast use another, parallel MAPK pathway to initiate haploid cell fusion, or mating. This pathway is activated by pheromone binding to a GPCR to initiate exchange of GDP for GTP in the Gα subunit (Gpa1) and subsequent dissociation of Gα from Gβγ. Gβγ activates a MAPKKK (Ste11, shared by the HOG pathway), a MAPKK (Ste7) and a MAPK (Fus3, or Kss1). Once activated, Fus3 promotes transcription of genes to initiate cell mating [28]. Fus3 is the yeast ortholog of mammalian ERK1 and ERK2 [29–32].

In the present study, we use yeast as a model system to investigate how crosstalk regulates G protein signaling during osmotic stress. We have shown previously that osmotic stress dampens the pheromone response pathway, and does so by Hog1-dependent and Hog1-independent mechanisms [33]. We have also shown that glucose limitation dampens the pheromone response pathway, and does so by reducing intracellular pH [34]. The increase in proton concentration is detected by the G protein directly, resulting in increased phosphorylation of Gpa1 and a dampened mating signal. Additionally, we have identified a family of three kinases (Elm1, Sak1, and Tos3) and a PP1 phosphatase complex (Reg1/Glc7) as the molecular machinery responsible for phosphorylating and dephosphorylating Gpa1 [21]. We show here that Gpa1 is likewise phosphorylated in response to osmotic stress, and that phosphorylation of Gpa1 requires the same protein kinases, but does not entail any changes in intracellular pH. In a search for alternative mediators of cross-pathway regulation, we conducted an unbiased metabolomics screen and found that 2-hydroxy branched chain amino acid metabolites are produced in a salt- and Hog1-dependent manner. Finally, we show that these metabolites are necessary and sufficient to promote Gpa1 phosphorylation and dampen downstream signaling. We propose that these metabolites represent a new class of second messengers of the stress-responsive HOG pathway.

## Results

### Gpa1 is phosphorylated in response to environmental stress

**Fig 1:**
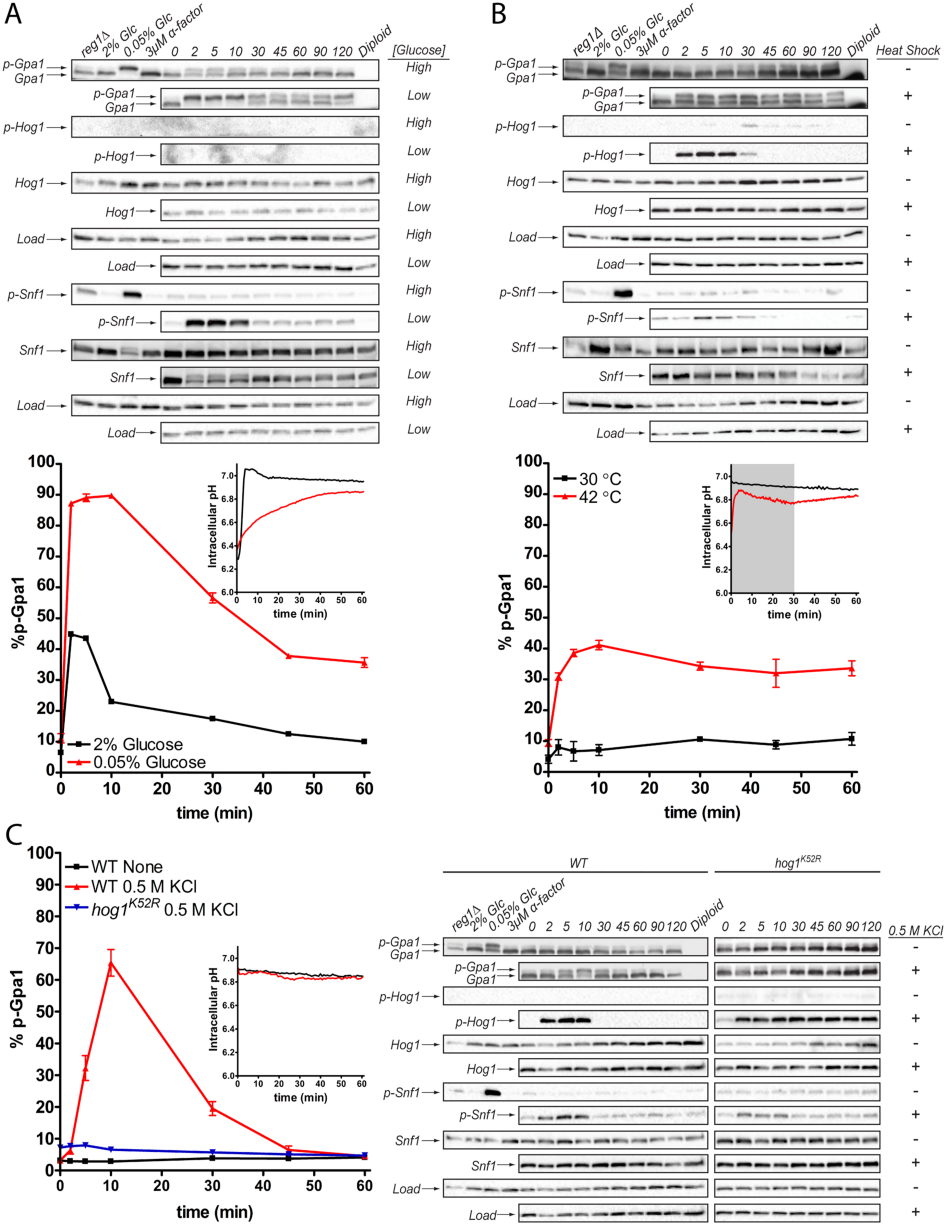
Phosphorylation of Gpa1 in response to osmotic stress occurs in a Hog1-dependent, pH-independent manner. Western blot analysis reveals that Gpa1 and Snf1 are phosphorylated (p-Gpa1 and p-Snf1) in response to (A) glucose (Glc) limitation (“High” = 2% glucose, “Low” = 0.05% glucose), (B) heat shock (42°C), or (C) osmotic stress (0.5 M KCl). Note that Hog1 is phosphorylated (p-Hog1) in response to heat shock or osmotic stress, but not glucose limitation. Intracellular pH (insets) decreases in response to glucose limitation or heat shock (shaded area), but not osmotic stress. Hog1 catalytic activity (*hog1*^*K52R*^) is required for phosphorylation of Gpa1 but not Snf1. Diploid, control cells lacking Gpa1. *reg1*Δ, control cells lacking Gpa1 phosphatase. Hog1, Snf1, and Load correspond to gels probed with Hog1, poly-His, and G6PDH antibodies, respectively. Data were quantified based on band intensity, and are presented as mean ± standard deviation, N = 3.

To understand how cells adapt to environmental stresses, we sought to identify conditions that impact pheromone signaling through the phosphorylation of Gpa1. We recently established that Gpa1 is phosphorylated by a family of three AMPK kinases (Elm1, Sak1, and Tos3), and dephosphorylated by the phosphatase complex Reg1/Glc7 [17, 21]. These proteins were previously shown to phosphorylate and dephosphorylate the yeast AMPK, Snf1 [35–37]. Snf1, is phosphorylated and activated in response to nutrient limitation, as well as heat shock, hyperosmotic shock, reactive oxygen species, ethanol, and changes in extracellular pH [38]. Accordingly, we asked whether the same environmental stressors would lead to phosphorylation of Gpa1. We treated wild-type cells with the indicated stressor in a 2-hour time-course (see Materials and methods), and analyzed cell lysates by western blot. As shown in Fig 1, Gpa1 and Snf1 were phosphorylated in all stress conditions tested (see also S1 Fig). However, among the stressors there were differences in the both the magnitude and duration of phosphorylation. In glucose-limiting conditions, approximately 90% of Gpa1 was phosphorylated by 2 minutes, with a gradual decline after 10 minutes (Fig 1A). Heat shock (at 42°C) also promoted rapid phosphorylation but slow dephosphorylation. Osmotic stress promoted slow phosphorylation, but fast dephosphorylation. Heat and osmotic stress also promoted the phosphorylation of Snf1, but the effects were comparatively weak and transient (Fig 1B and 1C) [38]. These data reveal that Gpa1, like Snf1, is phosphorylated in response to various stress signals. More broadly, the results indicate that physico-chemically distinct stimuli have a common ability to promote phosphorylation of two functionally distinct proteins, Snf1 and Gpa1.

It is well-established that the MAPK Hog1 is phosphorylated and activated in response to osmotic stress [22]. Hog1 is also activated by heat shock [39], cold stress [40], oxidative stress [41], and hypoxia [42]. Given that many of these conditions also lead to phosphorylation of Gpa1 and Snf1, we asked if Hog1 activation was required in either case. To this end, we replaced Hog1 with a mutant documented to lack catalytic activity, *hog1*^*K52R*^ [43], and then treated the cells with 0.5 M KCl. Whereas Snf1 phosphorylation was unperturbed, the phosphorylation of Gpa1 was almost completely abrogated in the *hog1*^*K52R*^ strain (compare Fig 1C, blue curve vs. red curve). It is unlikely that Hog1 phosphorylates Gpa1 directly, since the relevant site (Ser200) does not adhere to the MAPK consensus sequence (Ser/Thr-Pro) [17]. Thus, Hog1 catalytic activity is required for the salt-induced phosphorylation of Gpa1 but not Snf1. More broadly, these results implicate at least two distinct signaling pathways, and potentially two distinct second messengers, that mediate the response to osmotic stress.

One potential second messenger is pH. Indeed, it is well established that glucose limitation leads to a substantial decrease in intracellular pH (pH_i_) [44]. We have shown recently that Gpa1 is a pH sensor, and that pH-dependent changes in conformation result in phosphorylation of the protein [34]. Since other stressors trigger phosphorylation of Gpa1, we asked whether any of those conditions also cause a change in pH_i_. To that end we expressed the ratiometric fluorescent pH biosensor, pHluorin, in wild-type cells [34, 45, 46]. Consistent with earlier studies [34], we observed a decrease in pH_i_ from 7.0 to 6.4 upon glucose limitation (Fig 1A, inset). In contrast, cells subjected to osmotic stress exhibited no change in pH_i_ over the course of 60 minutes (Fig 1C and S1 Fig). These data indicate that low glucose and osmotic stress each promote Gpa1 phosphorylation, but glucose alone affects pH_i_. We therefore postulated the existence of an additional second messenger of the osmotic stress response.

### Global metabolomics analysis for second messengers of osmotic stress

**Fig 2:**
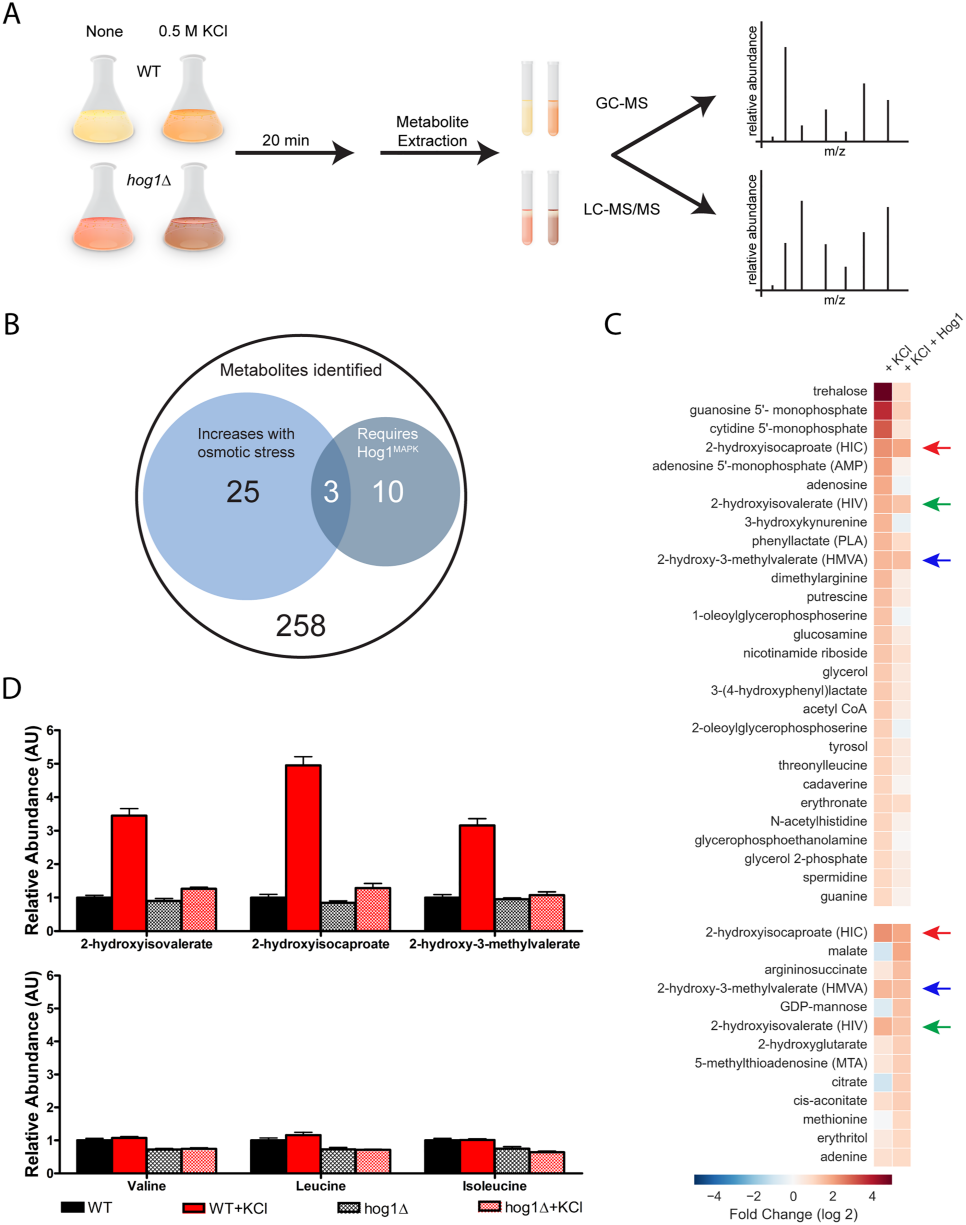
Global metabolomics analysis identifies candidate second messengers of osmotic stress. (A) Metabolites from wild-type and *hog1*Δ cells, untreated or treated for 20 minutes with 0.5 M KCl, were extracted and then analyzed by GC-MS and LC-MS/MS. (B) 296 unique metabolites were identified. Venn diagram of metabolites that increase >2-fold in response to osmotic stress (n = 28), in cells that express Hog1 (n = 13) or both (n = 3). (C) Heat map of metabolites that increase in salt-treated wild-type compared to unstressed wild-type cells (left column, top), and increase in salt-treated wildtype, but not salt-treated *hog1*Δ cells (right column, bottom). Colored arrows indicate 2-hydroxy carboxylic acid derivatives of the BCAAs valine (HIV, green), leucine (HIC, red), and isoleucine (HMVA, blue). (D, Top) Relative abundance of the three BCAA derivatives and (D, Bottom) their parent amino acids. Data presented as mean ± standard deviation, N = 5.

The data presented above reveal that osmotic stress has no effect on pH_i_, yet is a potent inducer of Gpa1 phosphorylation. To identify potential second messengers of osmotic stress, we conducted a global, unbiased metabolomics analysis [47]. Based on results from the Gpa1 phosphorylation experiments above, we sought to identify metabolites that increased with osmotic stress and did so in a Hog1-dependent manner. To this end, we subjected wild-type and Hog1-deficient yeast cells to 0.5 M KCl for 20 minutes and then analyzed cell extracts by LC-MS/MS and GC-MS (Fig 2A). This analysis identified 296 distinct entities representing each major class of biochemical molecules—amino acids, peptides, carbohydrates, lipids, nucleic acids, vitamins and cofactors, and xenobiotics (S1 Table). Consistent with past findings, we found that the osmolytes trehalose [48] and glycerol [49] were induced substantially (32-fold and 2.5-fold, respectively) (Fig 2C). Using a comparable (2-fold) cut off, we identified an additional 26 metabolites that increased in response to osmotic stress, and 13 that increased in the presence of Hog1. Of these, only three required osmotic stress and Hog1 together (Fig 2B and 2C, S2 Table): 2-hydroxyisovalerate (HIV), 2-hydroxyisocaproate (HIC), and 2-hydroxy-3-methylvalerate (HMVA). All three compounds are 2-hydroxy carboxylic acid derivatives of the branched-chain amino acids (BCAAs) valine, leucine, and isoleucine, respectively (Fig 2C and 2D). Thus, our analysis points to 2-hydroxy BCAA derivatives as candidate second messengers of osmotic stress.

### Branched-chain aminotransferases are required for proper osmotic stress signaling

**Fig 3:**
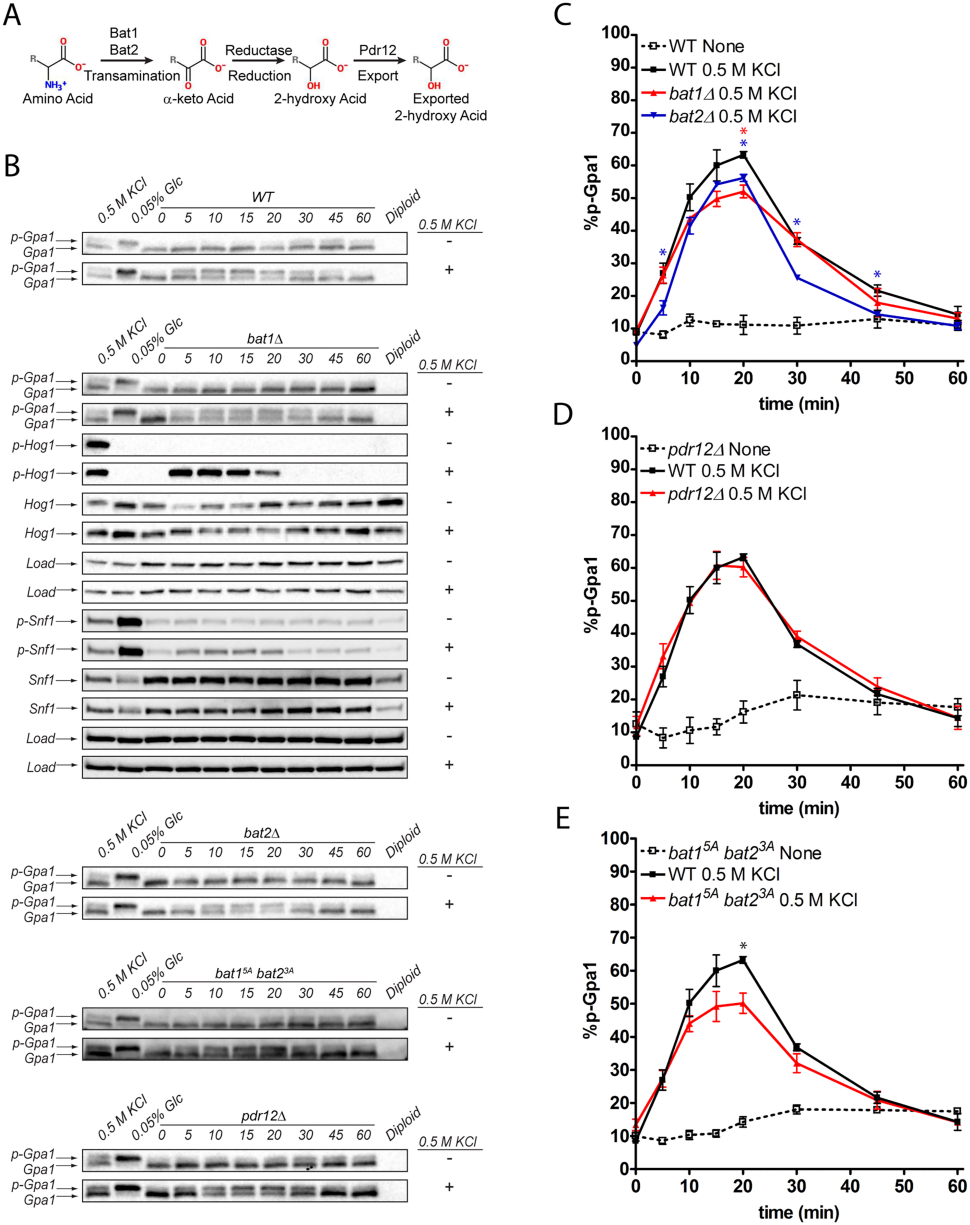
BCAA derivatives are necessary for a full response to osmotic stress. (A) BCAAs are converted to 2-keto acids by the BCAA transaminases, Bat1 and Bat2. The 2-keto acids are subsequently reduced to the 2-hydroxy acids, and ultimately exported by the fusel acid transporter, Pdr12. (B, C) Genetic ablation of *BAT1* or *BAT2*, or (B, E) loss of MAPK phosphorylation consensus sites (*bat1*^5A^ *bat2*^3A^) leads to reduced Gpa1 phosphorylation. (B, D) Genetic ablation of *PDR12* does not affect Gpa1. Data presented as mean ± standard deviation, * indicates p ≤ 0.05, N = 3.

Our metabolomics study demonstrated that BCAA derivatives are produced in response to osmotic stress, and that their production requires Hog1 (Fig 2D). In principle, deleting Hog1 could alter the production of additional second messengers that may not have been detected in our metabolomics screen. However, as BCAA derivatives were the most robustly increased metabolites that met our criteria for osmotic stress, we examined the consequences of disrupting BCAA catabolism through the so-called Ehrlich pathway in yeast [50]. The first step in the Ehrlich pathway is transamination to a 2-keto acid by the branched-chain amino acid transaminases, Bat1 and Bat2. The second step is decarboxylation of the 2-keto acid to an aldehyde, which is subsequently converted to a fusel acid or fusel alcohol. The BCAA derivatives identified here retain the same carbon skeleton as the parent amino acids, suggesting the existence of an alternative metabolic route consisting of transamination followed by reduction to the 2-hydroxy acid (Fig 3A). Products of the Ehrlich pathway are exported from the cell by the ABC transporter Pdr12 [50, 51].

To test whether BCAA derivatives are required for phosphorylation of Gpa1 and/or Snf1, we deleted the *BAT1* and *BAT2* genes individually (Fig 3B). After osmotic stress, we observed a modest, but significant reduction in maximal phosphorylation of Gpa1 in the *bat1*Δ and *bat2*Δ mutants, as compared to wild-type cells (Fig 3B and 3C). As expected, Snf1 phosphorylation was unaffected (Fig 3B). Cells harboring deletion of both *BAT* genes are reported to be viable [52, 53]; however in our hands, *bat1*Δ*bat2*Δ double mutants arose at a lower-than-predicted frequency after tetrad dissection and likely harbored suppressor mutations. As an alternative approach, we attempted to use a tetracycline-repressible *BAT1* in a *bat2*Δ background. However, the doxycycline used to repress *BAT1* expression also promoted the phosphorylation of Gpa1. Gpa1 phosphorylation was unaffected by loss of the transporter gene *PDR12* (Fig 3D), suggesting other routes of removal or of further metabolism. Together these results indicate that either Bat1 or Bat2 is necessary for cell viability. Both proteins, as well as their catalytic products, are necessary for a full response to osmotic stress.

### MAPK-dependent phosphorylation of branched-chain amino acid transaminases does not affect Gpa1 phosphorylation

Our results indicate that Hog1 activity and BCAA catabolism are both needed for a full response to osmotic stress. In particular, we have shown that osmotic stress-dependent Gpa1 phosphorylation is reduced in mutants lacking Bat1 or Bat2, and is eliminated in cells lacking Hog1 catalytic activity. Based on these findings, we hypothesized that Hog1 phosphorylates one or more components of the BCAA pathway. Indeed, Bat1 has five MAPK consensus sites (S/TP), and Bat2 has three such sites. In support of our hypothesis, replacement of the MAPK consensus sites in Bat1 and Bat2 led to a significant reduction in Gpa1 phosphorylation (Fig 3B and 3E). However, there were no changes in the electrophoretic (phosphorylation-dependent) mobility of Bat1, Bat2, Bat1^5A^, or Bat2^3A^, either in the absence or presence of salt stress. There was also no effect of osmotic stress on Bat2^3A^ in cells lacking Bat1 (*bat1*Δ *bat2*^3A^) or Bat1^5A^ in the absence of Bat2 (*bat1*^5A^ *bat2*Δ) (S2 Fig). Taken together, these results suggest that Hog1 does not target the transaminases, and instead plays an indirect role in promoting the production of BCAA derivatives. That role could be to induce phosphorylation, or regulate the transcription, of some other component of the metabolic pathway. One potential target is the reductase(s) (as yet unidentified) that converts the 2-keto acid to the 2-hydroxy acid.

### Ectopic addition of BCAA derivatives promotes Gpa1 phosphorylation in the absence of osmotic stress

**Fig 4:**
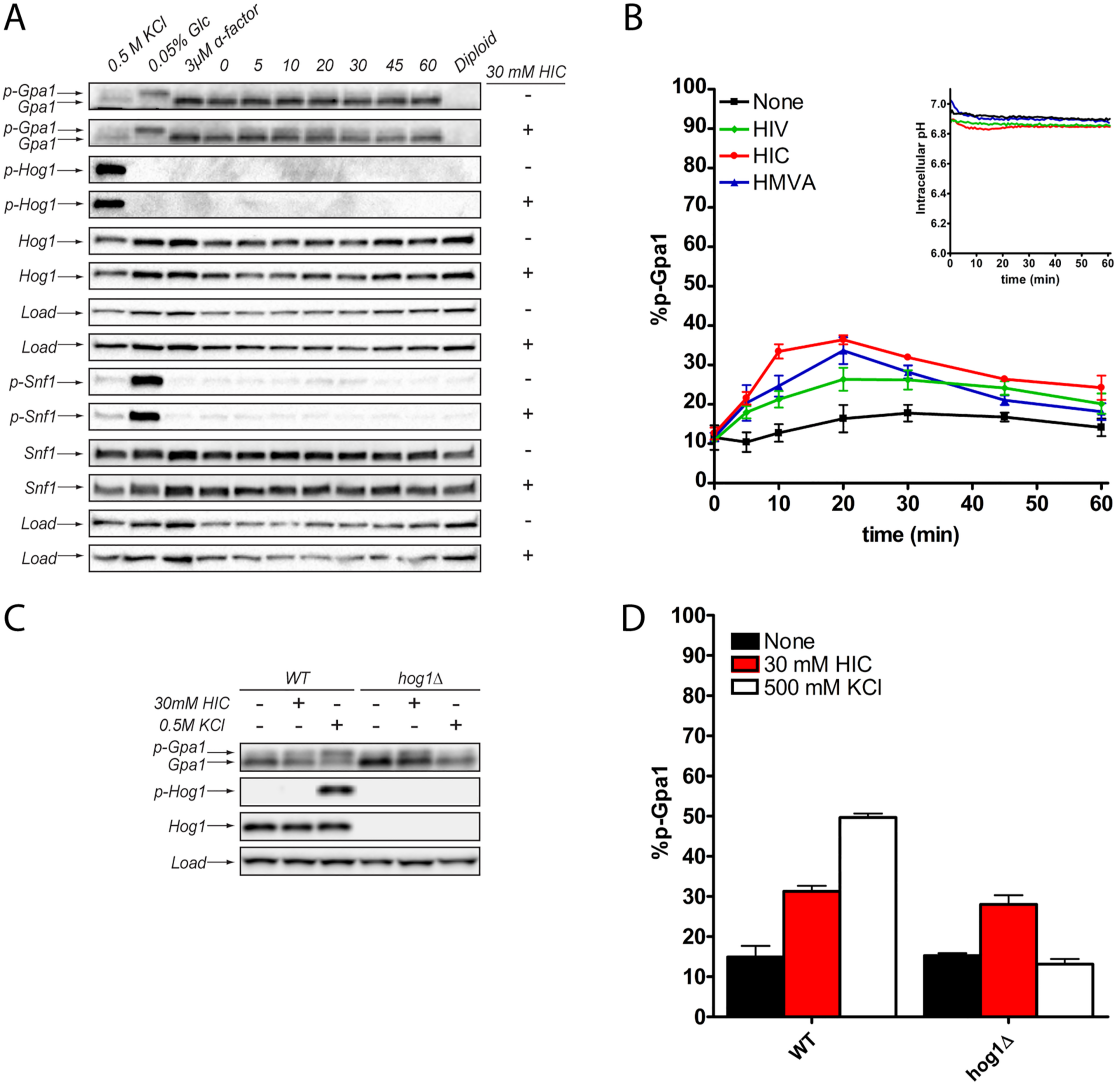
BCAA derivatives promote Gpa1 phosphorylation. (A) Ectopic addition of 2-hydroxyisocaproate (HIC) promotes phosphorylation of Gpa1 but not Hog1 or Snf1. (B) Ectopic addition of the BCAA derivatives promotes Gpa1 phosphorylation while intracellular pH is unaffected (inset). (C, D) Ectopic addition of HIC promotes Gpa1 phosphorylation in wild-type and Hog1-deficient cells. Osmotic stress promotes Gpa1 phosphorylation only in wild-type cells. Data presented as mean ± standard deviation, N = 3.

An intracellular second messenger should, by definition, be sufficient as well as necessary to evoke the response of the extracellular first messenger. Having demonstrated that BCAA derivatives are necessary for a full response to osmotic stress, we tested the ability of the BCAA derivatives to promote phosphorylation in the absence of salt stimulus. To better enable these compounds to traverse the cell membrane, we grew the cells at pH 5, which is closer to the pK_a_ of the metabolites. By favoring the protonated, uncharged species, the BCAA derivatives can more easily traverse the plasma membrane. Importantly, the lower external pH does not change the intracellular pH (Fig 4B, inset) [34]. Using this approach, we found that HIV, HIC, and HMVA promoted Gpa1 phosphorylation, but with varying efficacy. HIC showed the strongest effect while HIV had the weakest effect (Fig 4A and 4B). Addition of HIC (but not salt) to Hog1-deficient cells promoted the phosphorylation of Gpa1, consistent with the idea that BCAA derivative production is a consequence of Hog1 activation (Fig 4A, 4C and 4D). Snf1 was likewise unaffected, consistent with the idea that it is regulated by a distinct second messenger (Fig 4A). Taken together, these experiments indicate that BCAA derivatives are sufficient to promote the phosphorylation of Gpa1 and thus meet the criteria for second messengers of osmotic stress.

### BCAA derivatives do not bind directly to Gα proteins

**Fig 5:**
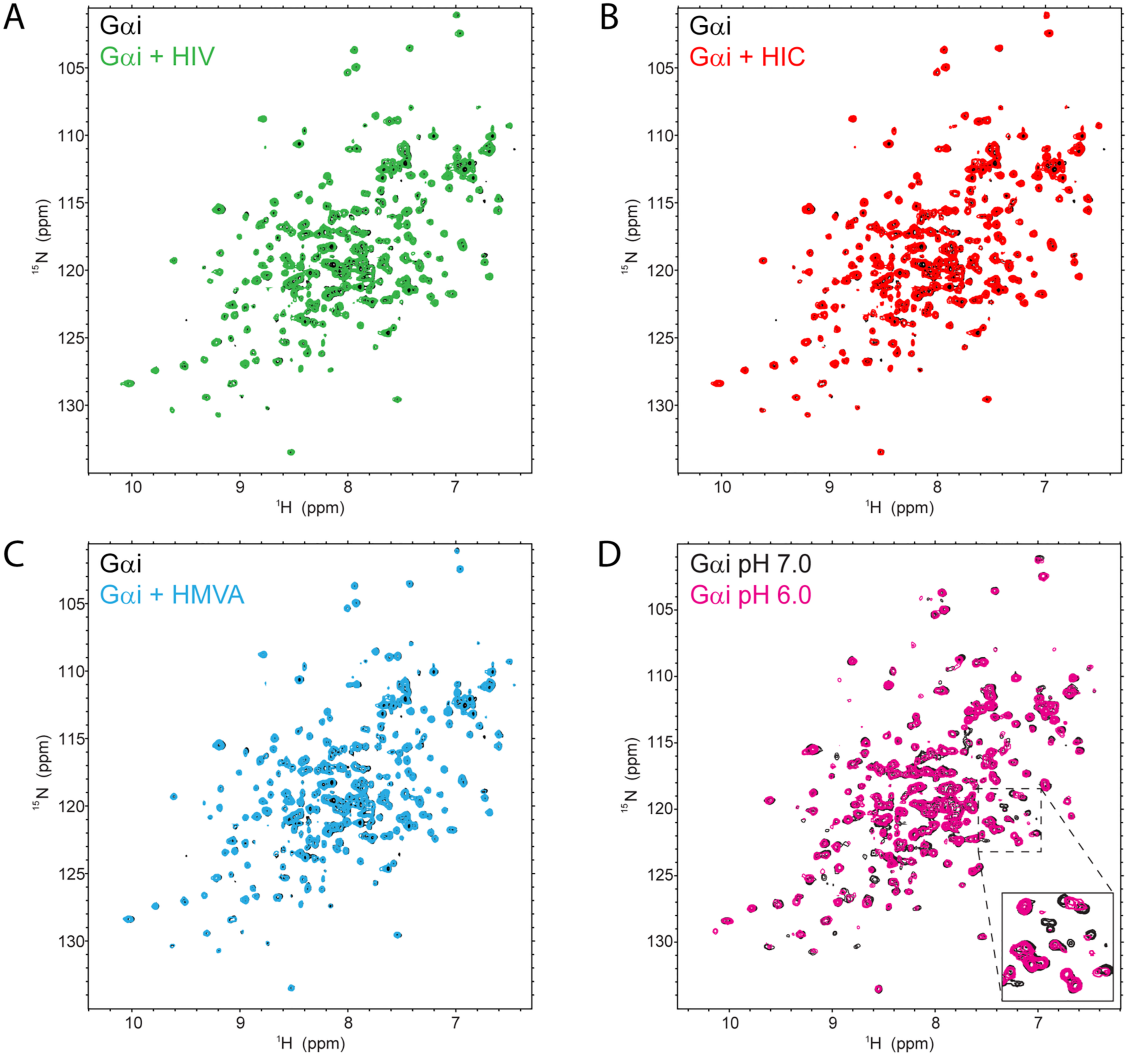
BCAA derivatives do not bind directly to Gα proteins. ^1^H-^15^N 2D HSQC NMR spectra of Gαi-GDP alone (black) or in the presence of 25-fold excess (A) HIV, (B) HIC, or (C) HMVA (color) reveal no discernable peak shifts. (D) Spectral overlay of Gαi-GDP at pH 6.0 (magenta) and pH 7.0 (black) is presented as a positive control. Inset, magnified view of a subset of resonances showing pH-dependent spectral changes.

Our results so far show that BCAA derivatives promote the phosphorylation of Gpa1. We next attempted to delineate the mechanism by which BCAA derivatives act. We demonstrated previously that protons interact directly with the G protein α subunit, causing a conformational change that promotes its phosphorylation. Moreover, the pH dependent change is conserved in yeast and human Gα proteins [34]. We hypothesized that BCAA derivatives might likewise act by binding to the Gα subunit. To test this we collected ^1^H-^15^N 2D heteronuclear NMR spectra of Gα, both in the absence and presence of BCAA derivatives. These spectra allow for the detection of protons directly bonded to a ^15^N, including both backbone and side-chain NH resonances. As an NH resonance can be detected for every residue, with the exception of proline, the spectrum contains a “fingerprint” of the protein backbone and allows perturbations resulting from interactions to be detected on a per-residue basis. This approach is widely considered as a definitive method for detecting low- to intermediate-affinity binding of ligands to proteins [54]. Accordingly, we acquired the NMR spectra of ^15^N-enriched Gαi in its GDP-bound state, alone or in the presence of a 25-fold excess of individual BCAA derivatives. As shown in Fig 5, there were no significant peak shifts when BCAA derivatives were present (Fig 5A–5C). As a positive control, we acquired NMR spectra of Gαi-GDP at pH 6 and at pH 7. As shown in Fig 5D, a substantial number of peaks were shifted at the lower pH, consistent with proton-dependent conformational changes in Gαi. These results indicate that BCAA derivatives likely act on another component of the G protein signaling pathway.

### The AMPK kinase Elm1 is required for phosphorylation of Gpa1 in response to osmotic stress and BCAA derivative production

**Fig 6:**
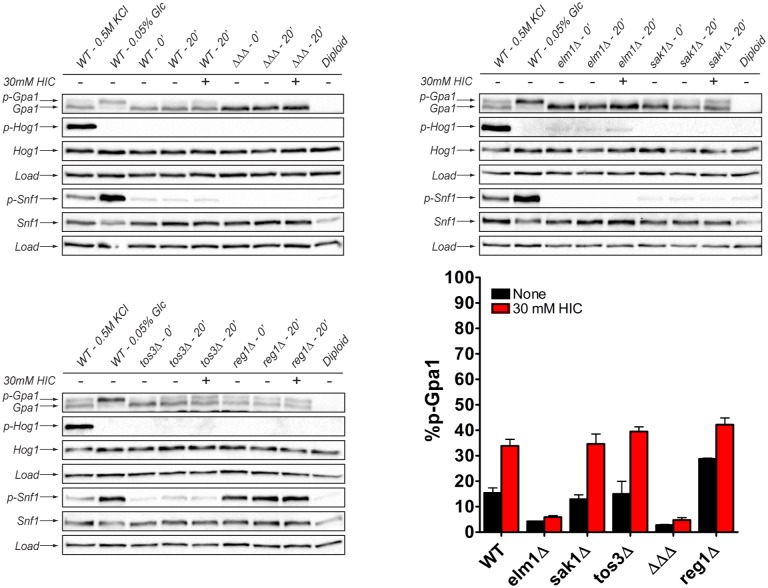
The AMPK kinase Elm1 phosphorylates Gpa1 upon BCAA derivative addition. Gpa1 phosphorylation after ectopic addition of 30 mM HIC is abrogated in cells lacking the AMPK kinase *ELM1*, or all three AMPK kinases (ΔΔΔ). Data presented as mean ± standard deviation, N = 3.

Gpa1 is phosphorylated by the AMPK kinases Elm1, Sak1, and Tos3. Whereas Elm1 phosphorylates Gpa1 in a cell-cycle-dependent manner [17], Sak1 is responsible for phosphorylation during glucose limitation [21]. Our data presented above indicate that Gpa1 is likewise phosphorylated in response to osmotic stress. To determine which, if any, of the AMPK kinases mediates the response to osmotic stress, we compared Gpa1 phosphorylation in cells lacking each of the three kinases, alone or in combination. As shown in S3A Fig, deletion of *ELM1* resulted in the greatest reduction of Gpa1 phosphorylation, while deletion of *SAK1* had a comparatively small effect. We then performed the same experiment using BCAA metabolites in place of osmotic stress. As with salt stimulation, HIC promoted the phosphorylation of Gpa1 in cells, and phosphorylation was diminished in the *elm1Δ* mutant (Fig 6 and S3B Fig). Taken together, these results indicate that both the primary messenger (osmotic stress) and the putative second messenger (the BCAA derivatives) act through Elm1. More broadly, these results confirm a fundamental difference between glucose- and salt-dependent changes in the cell. While both conditions lead to Gpa1 phosphorylation, they lead to the production of two distinct second messengers (protons and BCAA derivatives) and to phosphorylation by two distinct protein kinases (Sak1 and Elm1).

### Osmotic stress and BCAA derivatives dampen the pheromone response MAPK pathway

**Fig 7:**
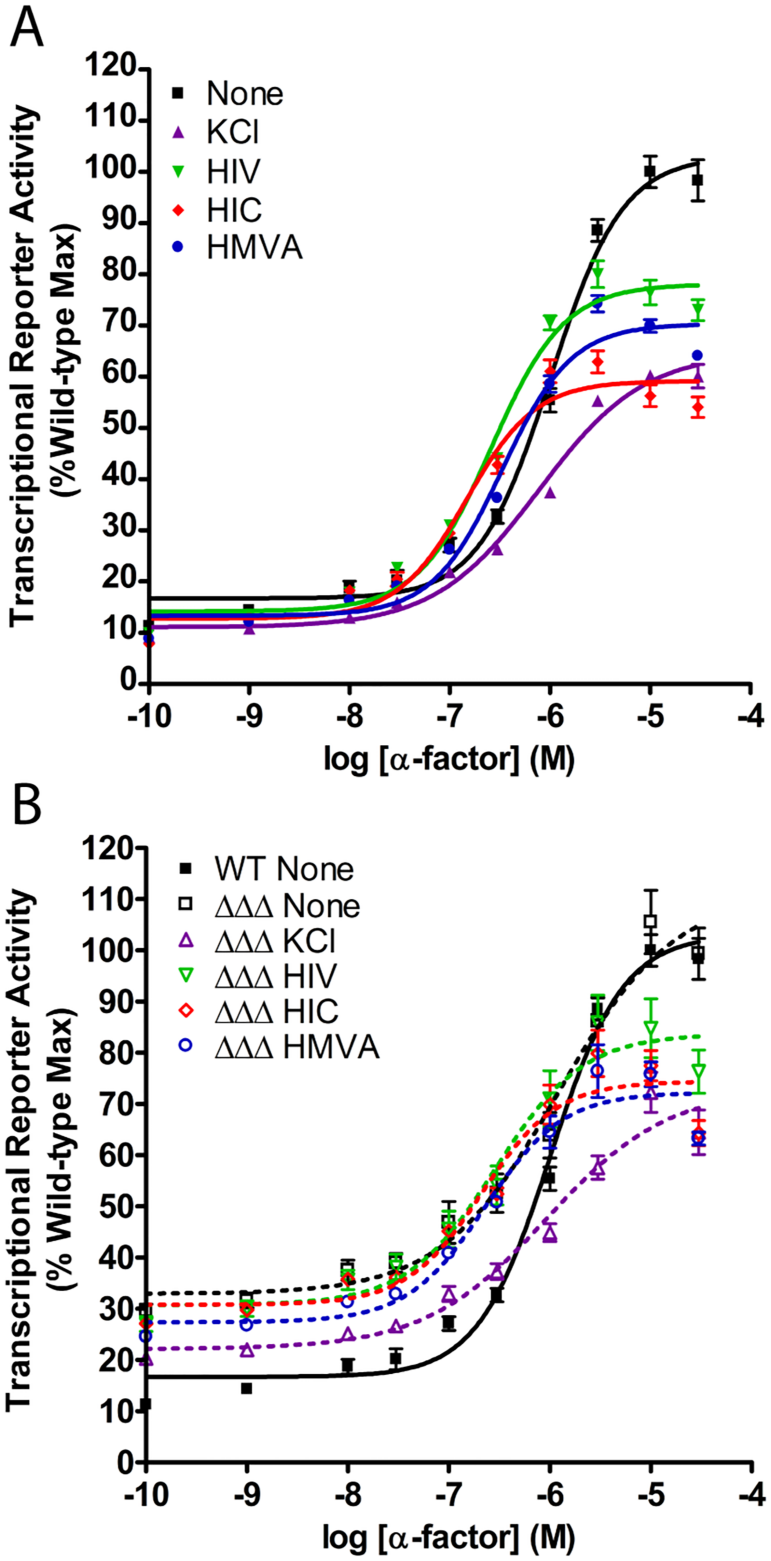
BCAA derivatives diminish MAPK-dependent gene transcription. (A) Addition of BCAA derivatives or KCl dampens α-factor pheromone-induced gene transcription (P_FUS1_-GFP). The dampening capacity of each BCAA derivative is correlated with Gpa1 phosphorylation (see Fig 4). (B) Genetic ablation of the AMPK kinases increases basal gene transcription, consistent with reduced Gpa1 phosphorylation [21]. Correspondingly, the kinase mutants abrogate any ability of the metabolites to suppress basal signaling and limit their ability to suppress pheromone signaling (41% reduction in wildtype vs. 26% reduction in the kinase mutant strain). Data are presented as mean ± standard deviation, N = 4.

We have shown that osmotic stress leads to a diminished pheromone response [33] and phosphorylation of the Gα protein (this work). Based on our model, the BCAA derivatives are responsible for many of the intracellular effects of osmotic stress signaling, including Gα phosphorylation. According to our proposed mechanism, the same metabolites should also dampen the response to pheromone. To test this hypothesis, we employed a transcriptional reporter assay using GFP under control of the *FUS1* promoter, which is specific to the pheromone response pathway [55]. We then measured fluorescence in response to increasing concentrations of the α-factor mating pheromone, alone or in combination with KCl or the BCAA derivatives. Consistent with previous reports [33], osmotic stress dampened the pheromone response by approximately 40%. Consistent with our present model, the addition of HIV, HIC, or HMVA also led to a diminished response of up to 40% (Fig 7A). The capacity of each BCAA derivative to dampen transcription correlated directly with its ability to promote Gpa1 phosphorylation (Fig 4B). Deletion of the Gpa1 kinases conferred an elevated signal at all but the highest concentrations of pheromone. At 10 μM pheromone the mutant strain was less sensitive to KCl and HIC (a reduction of 27% and 26%) compared to wild type (35 and 41%, respectively). At low and intermediate concentrations, the mutant strain was less responsive to salt and largely unresponsive to the BCAA derivatives (Fig 7B). Thus, BCAA derivatives are produced in response to an osmotic stress stimulus and, by every measure used, approximates the biochemical effects of salt on Gpa1. By these criteria the BCAA derivatives could function as second messengers of the osmotic stress response pathway and account for part of the osmotic stress response program.

## Discussion

Here, we present several novel features of the pheromone response pathway that we believe will be generally applicable to other MAPK signaling systems. First, we show that multiple environmental stressors lead to G protein phosphorylation. Phosphorylation of Gpa1 is accompanied by attenuated signaling through the effector MAPK, Fus3 [21, 33, 34]. Second, we show that many of these same stressors trigger the activation of another MAPK, Hog1. When Hog1 is activated, Fus3 signaling is inactivated. Third, we present the results of a comprehensive screen for small molecule metabolite second messengers, and show that 2-hydroxy BCAA derivatives are generated in response to osmotic stress and Hog1 activation. We show further that these metabolites are sufficient to trigger Gpa1 phosphorylation and a dampening of the Fus3 pathway. Finally, we show that the protein kinase Elm1 is required for phosphorylation of Gpa1 in response to osmotic stress and by addition of the metabolites. These processes are clearly distinct from those reported previously for glucose stress, which leads to a decrease in cellular pH, direct binding of protons to the Gα subunit, and direct phosphorylation of Gα by Sak1. While the target of the BCAA metabolites remains to be identified, we have largely excluded the kinase and Gα subunit substrate as candidates.

Based on our findings, we propose that BCAA metabolites represent a newly described “second messenger” of stress-activated signaling. The concept of second messenger signaling stems from the work of Earl Sutherland in 1957 [56] when he discovered that the activity of liver phosphorylase is stimulated indirectly by hormones, requiring a “heat-stable factor” that was later identified as cAMP [57]. That work established a paradigm of cell signaling whereby a first messenger (e.g., hormone or neurotransmitter) activates a receptor on the cell surface (canonically a GPCR) and activation of an intracellular effector protein that produces the second messenger molecule. This process serves to greatly amplify the intracellular response since activation of one receptor can lead to the production of multiple second messenger molecules. Since the discovery of cAMP, several other second messengers have been identified, including cGMP [58], inositol trisphosphate [59, 60], diacylglycerol [61], and calcium [62, 63]. Each of these molecules was painstakingly identified through rudimentary biochemical methods. With advances in metabolomics technologies however, we now have the ability to examine a broad complement of small molecules in a single experiment.

In yeast, BCAAs are catabolized through the Ehrlich pathway. The end products of this pathway are fusel alcohols or fusel acids [50]. Much like the catabolism of BCAAs by the Ehrlich pathway in yeast, BCAAs in mammals are metabolized to 2-keto acids by the branched-chain amino acid transaminases. The 2-keto acids primarily undergo oxidative decarboxylation by branched-chain keto acid dehydrogenase to yield substrates for further oxidation and generation of anaplerotic compounds for the TCA cycle [64]. However, the molecules characterized here appear to have undergone an alternative route, wherein 2-keto acids are reduced to form 2-hydroxy acids. Excess levels of 2-hydroxy acids are found in human patients with maple syrup urine disease, also known as branched-chain ketoaciduria. This is an autosomal recessive disorder caused by a deficiency in dehydrogenase activity. Without this enzyme, 2-keto acids accumulate and are shunted towards formation of 2-hydroxy acids [65, 66]. Accumulation of 2-keto and 2-hydroxy acids often results in brain damage due to impaired neurotransmitter function caused by inhibition of glutamate uptake [67, 68], and neuronal energy metabolism dysfunction [69, 70]. Although 2-hydroxy acids are produced, the accumulation of BCAAs and 2-keto acids seems to have the greater impact on the pathophysiology of maple syrup urine disease [71].

Previously we showed that osmotic stress dampens and delays the mating pheromone response in yeast [33]. Here we describe potential mechanisms of this cross-pathway regulation. While our analysis focused on yeast, several tissues routinely experience osmotic stress, and can develop disease if osmoregulation is impaired. For example, osmotic stress can promote dry eye disease [72] and diabetic retinopathy [73]. High osmolarity in the vasculature can lead to hypertension [74] and a hyperosmolar hyperglycemic state in diabetics [75]. Importantly, BCAA metabolism is also conserved in humans [76]. Reduced levels of the BCAAs are observed in heart failure, sepsis, trauma, and burn injury [77]. Moreover, a reduction in the expression of branched-chain amino acid transaminase and keto acid dehydrogenase, as well as an increase in the levels of 2-keto acids, have been identified as hallmarks of heart failure [78]. However the connection between osmotic stress signaling and BCAA metabolism is not clearly understood. Collectively, these examples highlight the need for a more complete understanding of the osmotic stress response and of BCAA metabolism.

In summary, we identified 2-hydroxy BCAA derivatives as candidate second messengers of the osmotic stress pathway. As second messengers, these molecules are likely used to amplify the osmotic stress response and coordinate responses to hormones and neurotransmitters. A challenge for the future is to determine the mechanism by which Hog1 (or p38) promotes BCAA derivative accumulation, their cellular target(s) in both yeast and humans, as well as their potential as lead molecules for pharmacological control of the stress response in a mammalian system.

## Materials and methods

### Strains and plasmids

All strains (S3 Table) were generated from the BY4741 wild type strain (*MAT***a** *his3*Δ*1 leu2*Δ*0 met15*Δ*0 ura3*Δ*0*) [79]. Gene deletion strains were generated by homologous recombination of PCR-amplified drug resistance genes from the pFA6a-KanMX6 [80] or pFA6a-hphMX6 plasmids [81], with flanking sequence homologous to the gene of interest [82], or by the delitto perfetto method, leaving no selection marker [83]. Similarly, Flag-tagged strains were generated by homologous recombination of the PCR-amplified cassette from pFA6a-6xGly-3xFlag-HIS3MX6 [84], with flanking sequence homologous to either side of the stop codon of the gene of interest. Bat1^5A^ Bat2^3A^ non-phosphorylatable mutants were generated using the delitto perfetto method. *BAT1* was replaced with the counter selectable marker and reporter gene cassette and then with synthesized *bat1*^5A^. The same steps were then used to replace *BAT2* with *bat2*^3A^. All cells were grown at 30°C unless otherwise noted.

The pRS426-P_FUS1_-YeGFP3 plasmid was generated by subcloning the YeGFP3 gene [85] under control of the yeast *FUS1* promoter from pDS30 (from Daria Siekhaus, University of California, Berkeley) [86] into pRS426 [87], by digestion with BamHI and XhoI, and ligation of gel-purified products. pYEplac181-pHluorin *(2μ*, *amp*^*R*^, *LEU2*^+^) was the gift of Rajini Rao (Johns Hopkins University) (S4 Table) [34, 45, 46].

### Environmental stress time courses

Cells were grown to saturation overnight in SCD medium, diluted to OD_600_ = 0.10, grown to OD_600_ ~0.6–0.8, then diluted again and grown to OD_600_ ~1.0. One third volume of SCD containing 3x stress stimulus was added to the experimental cell cultures. Control samples were mixed with 1/3 volume of SCD alone. Aliquots were collected at the times indicated, mixed 19:1 with 6.1 N trichloroacetic acid (TCA) and placed on ice. Cell pellets were collected by centrifugation at 1962 x g for 2 minutes, and resuspended in 10 mM NaN_3_. Cells were collected by centrifugation at 16,060 x g for 1 minute, the supernatant was removed, and cell pellets were stored at -80°C until use.

Heat shock experiments were carried out by growing cells as indicated above, then transferring the cultures to a 42°C water bath incubator/shaker and adding 1/3 final volume of SCD medium pre-warmed to 42°C. Control samples were mixed with 1/3 volume of media at 30°C.

For glucose limitation experiments, wild-type cells were grown as above to OD_600_ ~0.8, collected by centrifugation at 1962 x g for 2 minutes, resuspended with one-quarter volume of glucose-free SC medium, centrifuged again and resuspended in the original volume of SCD medium containing either 2% or 0.05% glucose. Note that the centrifugation step leads to partial and transient Gpa1 phosphorylation (Fig 1A, 2% Glucose curve).

### Cell lysis and protein quantification

Cell pellets were thawed on ice, and resuspended in ice cold TCA buffer (10 mM Tris-HCl, pH 8.0, 10% TCA, 25 mM ammonium acetate, 1 mM ethylenediaminetetraacetic acid). Cells were vortexed for 10 minutes, then collected by centrifugation at 16,060 x g for 10 minutes at 4°C. Pellets were reconstituted in resuspension buffer (100 mM Tris-HCl, pH 11.0, 3% sodium dodecyl sulfate (SDS)), heated at 99°C for 10 minutes, cooled to room temperature for 10 minutes, and centrifuged at 16,060 x g for 1 minute. Lysates were transferred to new tubes and 5 μL was used in a Bio-Rad DC Protein Assay (Bio-Rad #5000112), carried out according to the manufacturer’s protocol, and compared against a bovine serum albumin standard curve. Lysates were normalized to 2 μg/μL with resuspension buffer and 6x SDS sample buffer (350 mM Tris-HCl, pH 6.8, 30%(v/v) glycerol, 10%(w/v) SDS, 600 mM dithiothreitol, 0.012%(w/v) bromophenol blue), and used immediately or stored at -80°C.

### Immunoblotting

Cell lysates were heated at 99°C for 10 minutes, then 40 μg of protein was loaded onto 10% SDS-PAGE gels. Gels were then run in SDS electrophoresis buffer (25 mM Tris base, 20 mM glycine, 0.1%(w/v) SDS) at room temperature for 20 minutes at 20 mA/gel after which, current was increased to 25 mA/gel for 110 minutes. Electrophoresed proteins were then transferred to nitrocellulose membranes at 100 V for 90 minutes at 4°C in transfer buffer (20% methanol, 25 mM Tris base, 200 mM glycine). Membranes were blocked in TBS-T (100 mM Tris Base, pH 7.5, 150 mM NaCl, 0.1% Tween-20) containing 5% (w/v) milk and 10 mM NaN_3_ for 1 hour unless otherwise indicated. Western blots were probed with antibodies raised against Gpa1 (in-house rabbit polyclonal antibody, 1:1,000 ratio) [88], phospho-Snf1 (phospho-AMPKα (Thr172) 40H9 Rabbit mAb, Cell Signaling Technology #2353, 1:2,000 ratio), Snf1 (poly histidine HIS-1 mouse mAb, Sigma-Aldrich #H1029, 1:3,000 ratio), Hog1 (Santa Cruz Biotechnology #sc-6815, 1:500 ratio), phospho-Hog1 (phospho-p38 MAPK (Thr180/Tyr182) 28B10 Mouse mAb, Cell Signaling Technology #9216, 1:500 ratio), and Glucose-6-phosphate dehydrogenase as a loading control (G6PDH, Sigma # A9521, 1:50,000 ratio). Blots were incubated with primary antibodies for 1 hour to overnight, washed 3 x 5 minutes with TBS-T, then incubated with horseradish peroxidase-conjugated secondary antibodies raised against rabbit (Bio-Rad #1662408), mouse (Bio-Rad #1721011), or goat (Santa Cruz Biotechnology #sc-2768) at a 1:10,000 ratio in TBS-T containing 5% (w/v) milk, and washed 3 x 5 minutes with TBS-T. Blots were imaged on a Bio-Rad ChemiDoc MP imaging system after a 5 minute incubation with Clarity ECL Western Blotting Substrate (Bio-Rad #1705061).

### Intracellular pH measurements

Wild type yeast were transformed with plasmid pYEplac181-pHluorin [34, 45, 46] and grown in SCD-Leu medium. For cells treated with BCAA derivatives (30 mM) the medium was titrated to pH 5.0 with HCl. Experiments and pH_i_ calculations were carried out as in [34] using the indicated stressor or metabolite at 3x stock concentration.

### Metabolomics

Wild type and *hog1*Δ cells were grown to saturation overnight, diluted to OD_600_ = 0.10 grown to OD_600_ ~0.6, diluted again to OD_600_ = 0.00075, incubated overnight to OD_600_ ~0.9. Cultures were then split in half and grown to OD_600_ ~1.0 and mixed 1:4 with SCD or SCD plus 2.5 M KCl. After 3 minutes the cultures were transferred to 250 mL conical bottles (Corning #430776) and centrifuged for 3 minutes at 1819.3 x g in a Sorvall RC3C Plus centrifuge using an H6000A swinging bucket rotor. After aspirating the supernatant the cell pellets were snap-frozen in place with liquid nitrogen and stored at -80°C. The cells were exposed to KCl for a total of 20 minutes. Frozen pellets were submitted to Metabolon, Inc. for GC-MS and LC-MS/MS analysis of metabolites (see S1 Methods).

### NMR spectroscopy

For NMR measurements, ^15^N-enriched Gαi-Δ31 produced as in [89] was exchanged into NMR buffer (20 mM sodium phosphate, pH 7.0, 50 mM NaCl, 2 mM MgCl_2_, 200 μM GDP, 5% D_2_O). Each NMR sample contained 50 μM Gαi-Δ31 and 1.25 mM ligand. NMR spectra were acquired at 25°C on a Bruker Avance 850 NMR spectrometer. Two-dimensional ^1^H–^15^N HSQC experiments were recorded with 1024 and 128 complex points in the direct and indirect dimensions, respectively, 44 scans per increment and a recovery delay of 1.0 seconds. Spectral widths used were 13586.957 Hz (^1^H) and 3015.682 (^15^N) Hz. Spectra were processed and analyzed using NMRPipe (NIDDK, NIH) and Sparky (UCSF).

### Transcriptional reporter assay

Four colonies of the same strain transformed with plasmid pRS426-P_FUS1_-YeGFP3 and one colony of the untransformed background strain (to use for background fluorescence subtraction) were grown to OD_600_ ~1.0. Samples were added in duplicate to black clear-bottomed 96-well plates containing 10x stocks of serially diluted α-factor mating pheromone ranging in concentration from 1x10^-4.5^ M to 1x10^-10^ M prepared in sterile water, and 5x stocks of stimulus solution prepared in growth medium. The OD_600_ for each well was measured for cell number normalization. After 3 hours, GFP fluorescence was measured at an excitation wavelength of 485 nm, and emission wavelength of 538 nm, using a cutoff of 530 nm, in a Molecular Devices Spectramax M5 plate reader. For data presentation, raw fluorescence values from each well were normalized to the number of cells in that well (represented by the OD_600_) using the shorthand Taylor Series $\frac{1}{1+x}$ where *x* = OD_600_. Normalized values of each technical duplicate were averaged, and normalized values from the background strain (containing no fluorescence reporter) were subtracted. Finally, each well was normalized as a percent to the average maximum fluorescence value in the α-factor treated positive control. Dose-response curves were fitted using a nonlinear Boltzmann function.

### Statistical analysis

All data are reported as mean ± the standard deviation. Statistical significance was determined by an unpaired two-sided student’s t-test. In all cases, a p-value ≤ 0.05 was considered to be statistically significant.

## Supporting information

S1 Fig Additional stress conditions promote Gpa1 phosphorylation with differing effects on intracellular pH. In addition to salt, heat and glucose stress, Gpa1 is phosphorylated in response to (A) non-ionic osmotic stress, (B) oxidative stress, (C) ethanol stress, and (D) alkaline pH. (A-D, insets) Intracellular pH decreases in response to oxidative- and ethanol stress, but not non-ionic osmotic stress, and increases in response to alkaline pH. Data presented as mean ± standard deviation, N = 3.
(TIF)

S2 Fig Bat1 and Bat2 phosphorylation and abundance are unaffected by osmotic stress. Phos-tag western blots of (A) Bat1-Flag and Bat1^5A^-Flag or (B) Bat2-Flag and Bat2^3A^-Flag reveal no detectable changes in phosphorylation after osmotic stress. (C) Western blot analysis of (C) Bat1-Flag or (D) Bat2-Flag reveals no change in abundance after osmotic stress. Putative non-phosphorylated (Bat1, Bat2), mono-phosphorylated (p-Bat1, p-Bat2) and dual phosphorylated (pp-Bat1, pp-Bat2) species are indicated. Band intensity for the corresponding phosphorylated (3p, 2p, and 1p) and unphosphorylated (0p) species was quantified by densitometry and plotted as a percentage of total abundance. WT, untagged control. Data presented as mean ± standard deviation, N = 3.
(TIF)

S3 Fig The AMPK kinase Elm1 phosphorylates Gpa1 upon osmotic stress or BCAA derivative addition. (A) Gpa1 phosphorylation after addition of 0.5 M KCl is diminished in cells lacking *ELM1* and abrogated in cells lacking all three AMPK kinases (ΔΔΔ). In contrast to Gpa1, phosphorylation of Snf1 requires Sak1 but not Elm1. (B) Gpa1 phosphorylation after ectopic addition of 30 mM HIC is abrogated in cells lacking the AMPK kinases *TOS3* and *ELM1* or *SAK1* and *ELM1*. Data presented as mean ± standard deviation, N = 3.
(TIF)

S1 Table Metabolomics study results. (XLSX)

S2 Table Metabolite fold-change values for Fig 2 heatmap. (DOCX)

S3 Table Yeast strains used in this study. (DOCX)

S4 Table Plasmids used in this study. (DOCX)

S1 Methods Supplemental materials and methods. (DOCX)
