# Supplementary figures and images for: Amino acid metabolites that regulate G protein signaling during osmotic stress
Source: PLoS Genet. 2017 May 30;13(5):e1006829. doi: 10.1371/journal.pgen.1006829 (PMC5469498; doi:10.1371/journal.pgen.1006829)

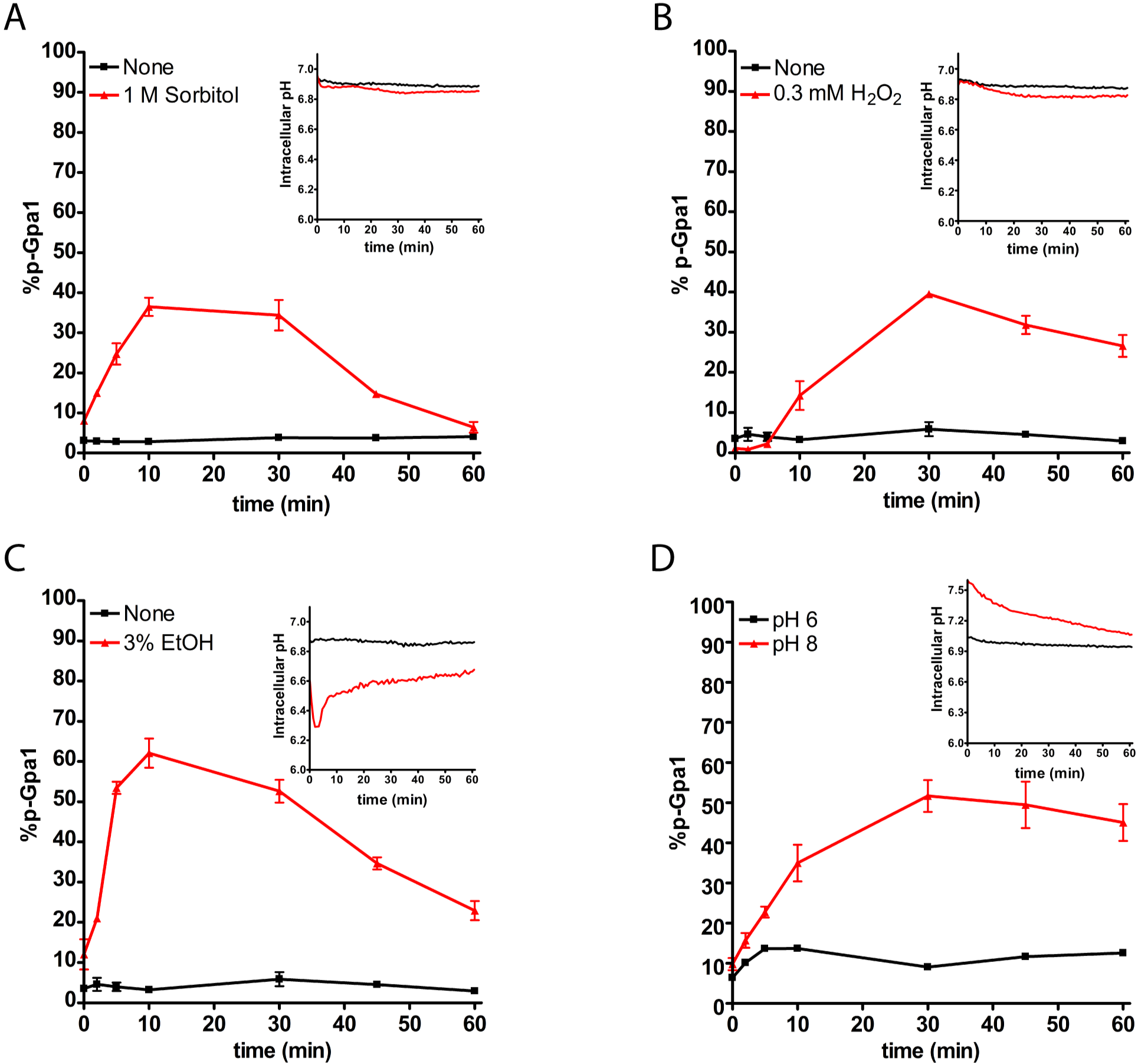

Supplement: S1 Fig — In addition to salt, heat and glucose stress, Gpa1 is phosphorylated in response to (A) non-ionic osmotic stress, (B) oxidative stress, (C) ethanol stress, and (D) alkaline pH. (A-D, insets) Intracellular pH decreases in response to oxidative- and ethanol stress, but not non-ionic osmotic stress, and increases in response to alkaline pH. Data presented as mean ± standard deviation, N = 3. (TIF) [file pgen.1006829.s001.tif]

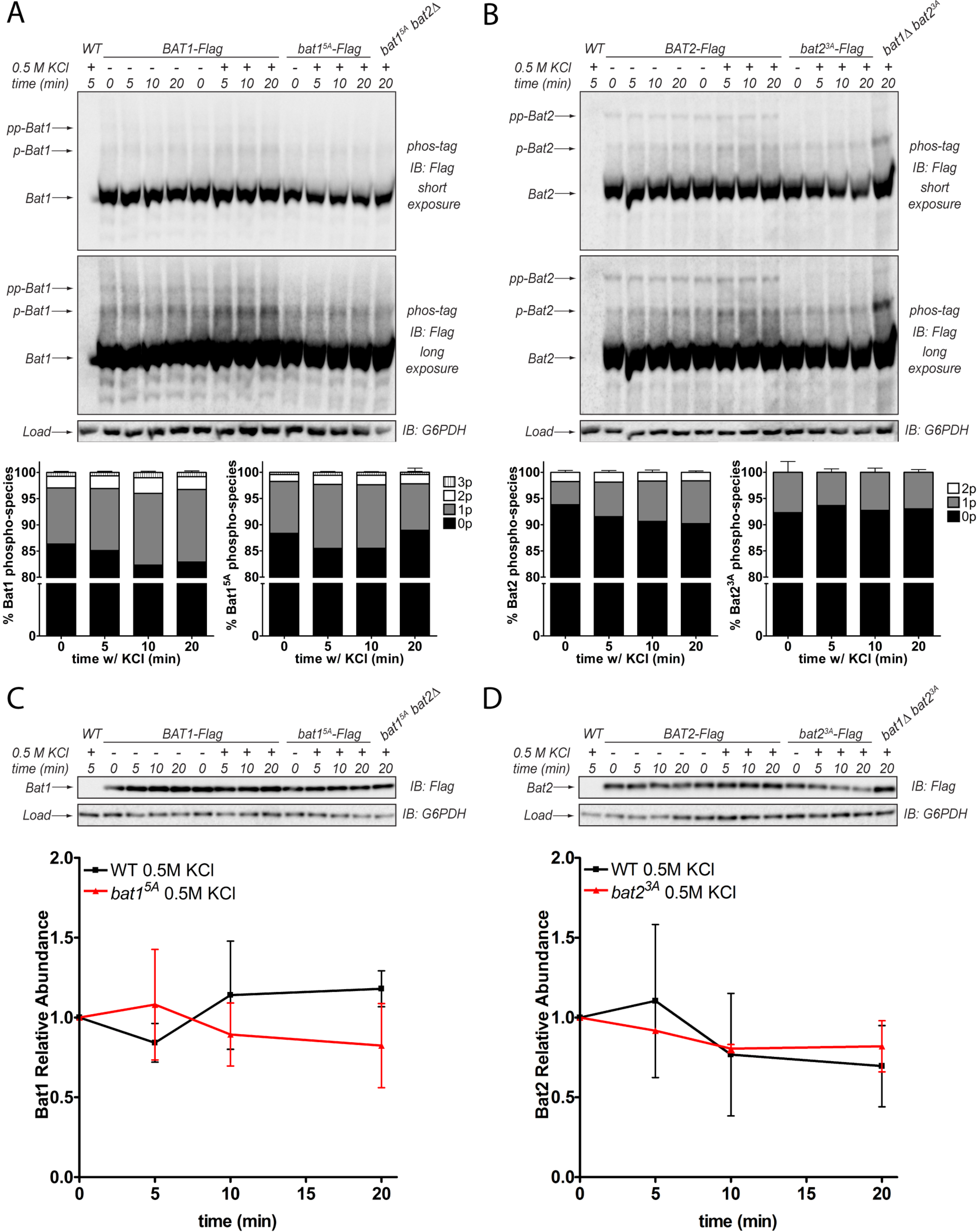

Supplement: S2 Fig — Phos-tag western blots of (A) Bat1-Flag and Bat15A-Flag or (B) Bat2-Flag and Bat23A-Flag reveal no detectable changes in phosphorylation after osmotic stress. (C) Western blot analysis of (C) Bat1-Flag or (D) Bat2-Flag reveals no change in abundance after osmotic stress. Putative non-phosphorylated (Bat1, Bat2), mono-phosphorylated (p-Bat1, p-Bat2) and dual phosphorylated (pp-Bat1, pp-Bat2) species are indicated. Band intensity for the corresponding phosphorylated (3p, 2p, and 1p) and unphosphorylated (0p) species was quantified by densitometry and plotted as a percentage of total abundance. WT, untagged control. Data presented as mean ± standard deviation, N = 3. (TIF) [file pgen.1006829.s002.tif]

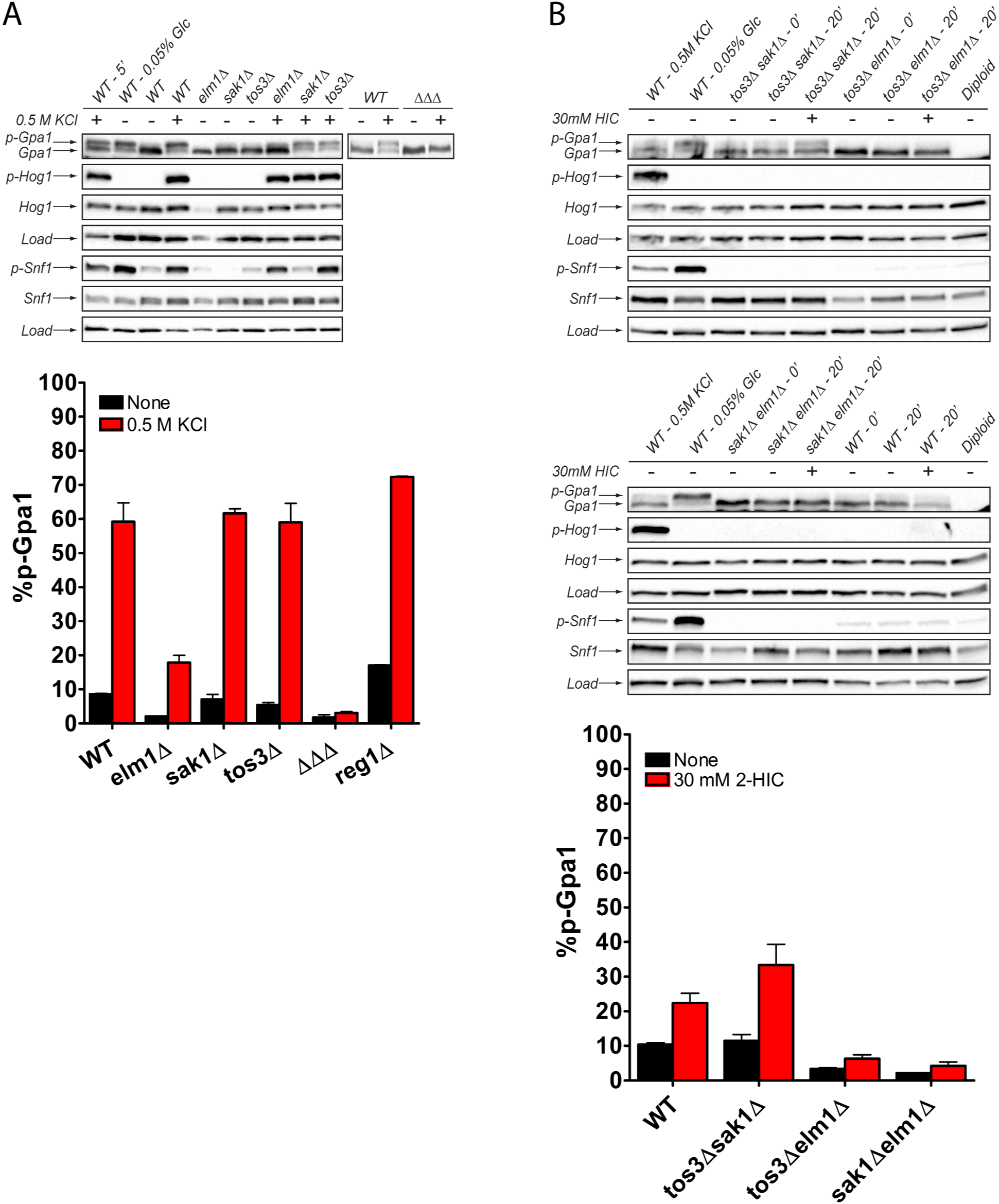

Supplement: S3 Fig — (A) Gpa1 phosphorylation after addition of 0.5 M KCl is diminished in cells lacking ELM1 and abrogated in cells lacking all three AMPK kinases (ΔΔΔ). In contrast to Gpa1, phosphorylation of Snf1 requires Sak1 but not Elm1. (B) Gpa1 phosphorylation after ectopic addition of 30 mM HIC is abrogated in cells lacking the AMPK kinases TOS3 and ELM1 or SAK1 and ELM1. Data presented as mean ± standard deviation, N = 3. (TIF) [file pgen.1006829.s003.tif]
