# Supplementary material for: Amino acid metabolites that regulate G protein signaling during osmotic stress
Source: PLoS Genet. 2017 May 30;13(5):e1006829. doi: 10.1371/journal.pgen.1006829 (PMC5469498; doi:10.1371/journal.pgen.1006829)
Supplement: S2 Table — (DOCX) [file pgen.1006829.s005.docx]

**Table S2. Metabolite Fold-change Values for Fig 2 Heatmap.**

| **Metabolite** | **Fold change with osmotic stress in wild-type vs. untreated wild-type** | **Fold change with osmotic stress in wild-type vs. *hog1Δ*** |
| --- | --- | --- |
| trehalose | **32.28** | 1.96 |
| guanosine 5'- monophosphate (5'-GMP) | **13.27** | 2.27 |
| cytidine 5'-monophosphate (5'-CMP) | **8.38** | 1.61 |
| **2-hydroxyisocaproate (2-HIC)** | **4.95** | **3.85** |
| adenosine 5'-monophosphate (AMP) | **4.24** | 1.20 |
| adenosine | **3.75** | 0.85 |
| **2-hydroxyisovalerate (2-HIV**) | **3.45** | **2.70** |
| 3-hydroxykynurenine | **3.23** | 0.78 |
| phenyllactate (PLA) | **3.21** | 1.96 |
| **2-hydroxy-3-methylvalerate (2-H3MP)** | **3.16** | **2.94** |
| dimethylarginine (SDMA + ADMA) | **3.05** | 1.32 |
| putrescine | **2.85** | 1.45 |
| 1-oleoylglycerophosphoserine | **2.8** | 0.88 |
| glucosamine | **2.63** | 1.41 |
| nicotinamide riboside* | **2.59** | 1.75 |
| glycerol | **2.51** | 1.47 |
| 3-(4-hydroxyphenyl)lactate | **2.5** | 1.52 |
| acetyl CoA | **2.42** | 1.35 |
| 2-oleoylglycerophosphoserine* | **2.26** | 0.81 |
| tyrosol | **2.23** | 1.49 |
| threonylleucine | **2.2** | 1.39 |
| cadaverine | **2.17** | 1.11 |
| erythronate* | **2.16** | 1.96 |
| N-acetylhistidine | **2.15** | 1.22 |
| glycerophosphoethanolamine | **2.11** | 1.04 |
| glycerol 2-phosphate | **2.07** | 1.32 |
| spermidine | **2.06** | 1.39 |
| guanine | **2.05** | 1.20 |
| **2-hydroxyisocaproate (2-HIC)** | **4.95** | **3.85** |
| malate | 0.53 | **3.85** |
| argininosuccinate | 1.54 | **2.94** |
| 2**-hydroxy-3-methylvalerate (2-H3MP)** | **3.16** | **2.94** |
| GDP-mannose | 0.62 | **2.78** |
| **2-hydroxyisovalerate (2-HIV)** | **3.45** | **2.70** |
| 2-hydroxyglutarate | 1.58 | **2.38** |
| 5-methylthioadenosine (MTA) | 1.57 | **2.33** |
| citrate | 0.5 | **2.33** |
| cis-aconitate | 1.84 | **2.33** |
| methionine | 0.95 | **2.08** |
| erythritol | 1.44 | **2.04** |
| adenine | 1.76 | **2.00** |
