# Supplementary material for: Amino acid metabolites that regulate G protein signaling during osmotic stress
Source: PLoS Genet. 2017 May 30;13(5):e1006829. doi: 10.1371/journal.pgen.1006829 (PMC5469498; doi:10.1371/journal.pgen.1006829)
Supplement: S3 Table — (DOCX) [file pgen.1006829.s006.docx]

**Table S3. Yeast Strains Used in this Study.**

| **Strain Name** | **Genotype** | **Source** |
| --- | --- | --- |
| BY4743 | *MAT****a****/α leu2Δ_0_/ leu2Δ_0_ LYS2/lys2Δ_0_ met15Δ_0_/MET15 his3-1/ his3-1 ura3Δ_0_/ ura3Δ_0_* | (Brachmann et al. 1998) |
| BY4741^a^ | *MAT****a*** *leu2Δ_0_ met15Δ_0_ his3-1 ura3Δ_0_* | (Brachmann et al. 1998) |
| *hog1Δ* | *MAT****a*** *hog1Δ_0_* | This study |
| *hog1^K52R^* | *MAT****a*** *hog1^K52R^* | (Hao et al. 2007) |
| *bat1Δ* | *MAT****a*** *bat1::KanMX4* | This study |
| *bat2Δ* | *MAT****a*** *bat2::HphMX6* | This study |
| *TetO_7_-BAT1-FLAG bat2Δ* | *MAT****a*** *CMV-tTA-URA3 KanMX4-TetO_7_-P_BAT1_ BAT1::6xGly-3xFLAG-HIS3MX6 bat2::HphMX6* | This study |
| *pdr12Δ* | *MAT****a*** *pdr12::KanMX4* | This study |
| *bat1^5A^ bat2^3A^* | *MAT****a*** *bat1^T68A, T101A, T175A, S186A, S339A^ bat2^T51A, T158A, S322A^* | This study |
| *BAT1-Flag* | *MAT****a*** *BAT1-6xGly-3xFLAG-HIS3MX6* | This study |
| *bat1^5A^-Flag* | *MAT****a*** *bat1^T68A, T101A, T175A, S186A, S339A^-6xGly-3xFLAG-HIS3MX6* | This study |
| *bat1^5A^-Flag bat2Δ* | *MAT****a*** *bat1^T68A, T101A, T175A, S186A, S339A^-6xGly-3xFLAG-HIS3MX6 bat2::HphMX6* | This study |
| *BAT2-Flag* | *MAT****a*** *BAT2-6xGly-3xFLAG-HIS3MX6* | This study |
| *bat2^3A^-Flag* | *MAT****a*** *bat2^T51A, T158A, S322A^-6xGly-3xFLAG-HIS3MX6* | This study |
| *bat1Δ bat2^3A^-Flag* | *MAT****a*** *bat1::KanMX4 bat2^T51A, T158A, S322A^-6xGly-3xFLAG-HIS3MX6* | This study |
| *elm1Δ* | *MAT****a*** *elm1::KanMX4* | (Clement et al. 2013) |
| *sak1Δ* | *MAT****a*** *sak1::KanMX4* | (Clement et al. 2013) |
| *tos3Δ* | *MAT****a*** *tos3::KanMX4* | (Clement et al. 2013) |
| *elm1Δ sak1Δ* | *MAT****a*** *elm1::URA3 sak1::KanMX4* | (Clement et al. 2013) |
| *elm1Δ tos3Δ* | *MAT****a*** *elm1::URA3 tos3::KanMX4* | (Clement et al. 2013) |
| *sak1Δ tos3Δ* | *MAT****a*** *sak1::URA3 tos3::KanMX4* | (Clement et al. 2013) |
| *elm1Δ sak1Δ tos3Δ* | *MAT****a*** *elm1Δ_0_ sak1::URA3 tos3::KanMX4* | This study |
| *elm1Δ sak1Δ tos3Δ* | *MAT****a*** *elm1Δ::URA3 sak1::LEU2 tos3::KanMX4* | (Clement et al. 2013) |
| *reg1Δ* | *MAT****a*** *reg1::KanMX4* | (Clement et al. 2013) |

^a^ All strains were derived from BY4741
