# Supplementary material for: Amino acid metabolites that regulate G protein signaling during osmotic stress
Source: PLoS Genet. 2017 May 30;13(5):e1006829. doi: 10.1371/journal.pgen.1006829 (PMC5469498; doi:10.1371/journal.pgen.1006829)
Supplement: S4 Table — (DOCX) [file pgen.1006829.s007.docx]

**Table S4. Plasmids Used in this Study.**

| **Plasmid Name** | **Descripion** | **Source** |
| --- | --- | --- |
| pYEplac181 | 2μ, amp^R^, LEU2+, P_TEF1_-pHluorin | (Isom et al. 2013) Dr. Rajini Rao,  Johns Hopkins |
| pRS426-P_FUS1_-YeGFP3 | 2μ, amp^R^, URA3+, P_FUS1_-YeGFP3 | This Study |
