# Supplementary material for: Amino acid metabolites that regulate G protein signaling during osmotic stress
Source: PLoS Genet. 2017 May 30;13(5):e1006829. doi: 10.1371/journal.pgen.1006829 (PMC5469498; doi:10.1371/journal.pgen.1006829)
Supplement: S1 Methods — (DOCX) [file pgen.1006829.s008.docx]

**Supplemental Materials and Methods**

*Phos-tag SDS-PAGE and Immunoblotting*

*Cell Lysis and Protein Quantification*

Frozen cell pellets were collected as described above. Cell pellets were thawed on ice, and resuspended in ice cold TCA buffer without EDTA (10 mM Tris-HCl, pH 8.0, 10% TCA, 25 mM ammonium acetate). Cells were lysed and lysate protein content was determined by the Bio-Rad DC Protein Assay as described above. Lysates were normalized to 1.5 μg/μL with resuspension buffer and 2x SDS sample buffer (500 mM Tris-HCl, pH 8.5, 20%(v/v) glycerol, 2%(w/v) SDS, 200 mM dithiothreitol, 0.01%(w/v) bromophenol blue), and used immediately or stored at -80°C.

*Phos-tag Bis-tris Acrylamide Gel Preparation and Transfer for Immunoblotting*

8% bis-tris SDS-PAGE gels containing 20 μM Phos-tag and 40 μM Zn(NO_3_)_2_ were prepared, run and transferred as described previously [1]. Briefly, protein samples were heated at 70°C for 10 minutes, then 15 μg of protein per lane was loaded onto Phos-tag gels. Gels were then run in phos-tag SDS-PAGE electrophoresis buffer (50 mM Tris base, 50 mM MOPS, 0.1%(w/v) SDS, 5mM sodium bisulfite, pH 7.2) at room temperature 150V for 90 minutes. Resolving layers were removed and equilibrated in transfer buffer (1x NuPAGE transfer buffer (Life Technologies # NP0006-1), 20%(v/v) methanol, 2.5 mM sodium pyrophosphate, 5 mM sodium bisulfite) for 15 minutes at room temperature with shaking to release phopho-proteins from Phos-tag. Electrophoresed proteins were then transferred to polyvinylidene difluoride (PVDF) membranes (Millipore # IPVH00010) 20 V for 20 hours at 4°C.

*Immunoblotting*

Membranes were blocked in TBS-T (100 mM Tris Base, 150 mM NaCl, 0.1% Tween-20, pH 7.5) containing 5% (w/v) milk and 10 mM NaN_3_ for 1 hour. Membranes were then probed for Flag-tagged proteins using the anti-Flag M2 primary antibody (Sigma # F3165) at a 1:10,000 ratio in blocking buffer, and Glucose-6-phosphate dehydrogenase as a loading control (G6PDH, Sigma # A9521, 1:50,000 ratio). Blots were incubated with primary antibodies for 1 hour, washed 3 x 5 minutes with TBS-T, then incubated with horseradish peroxidase-conjugated secondary antibodies raised against rabbit (Bio-Rad #1662408), or mouse (Bio-Rad #1721011) at a 1:10,000 ratio in TBS-T containing 5% (w/v) milk for 1 hour, and washed 3 x 5 minutes with TBS-T. Blots were imaged on a Bio-Rad ChemiDoc MP imaging system after a 5 minute incubation with Clarity ECL Western Blotting Substrate (Bio-Rad #1705061).

*Metabolomics Study*

*Experimental Design*

This 40 sample study used the yeast model system. The study had 8 groups powered at 5 samples per group with 4 treatment conditions: control (CTRL), salt (KCl), α-factor (α-F) and combined salt and α-factor (KCl+α).

*Sample Preparation*

Samples were stored at –70°C until processed. Sample preparation was carried out as described previously [2] at Metabolon, Inc. Briefly, recovery standards were added prior to the first step in the extraction process for quality control purposes. To remove protein, dissociate small molecules bound to protein or trapped in the precipitated protein matrix, and to recover chemically diverse metabolites, proteins were precipitated with methanol under vigorous shaking for 2 min followed by centrifugation. The resulting extract was divided into four fractions: one for analysis by ultra-high performance liquid chromatography-tandem mass spectrometry (UPLC-MS/MS; positive mode), one for analysis by UPLC-MS/MS (negative mode), one for analysis by gas chromatography–mass spectrometry (GC-MS), and one sample was reserved for backup.

Three types of controls were analyzed in concert with the experimental samples: samples generated from a pool of each experimental sample served as technical replicates throughout the metabolomics platform run; extracted water samples served as process blanks; and a cocktail of standards spiked into every analyzed sample allowed instrument performance monitoring. Instrument variability was determined by calculating the median relative standard deviation (RSD) for the standards that were added to each sample prior to injection into the mass spectrometers (median RSD = 5; n = 29 standards). Overall process variability was determined by calculating the median RSD for all endogenous metabolites (i.e., non-instrument standards) present in 100% of the pooled yeast technical replicate samples (median RSD = 9%; n = 296 metabolites). Experimental samples and controls were randomized across the platform run.

*Mass Spectrometry Analysis*

Non-targeted MS analysis was performed at Metabolon, Inc. Extracts were subjected to either GC-MS [3] or UPLC-MS/MS [2]. The chromatography was standardized and once the method was validated, and no further changes were made. As part of Metabolon’s general practice, all columns were purchased from a single manufacturer’s lot at the outset of experiments. All solvents were similarly purchased in bulk from a single manufacturer’s lot in sufficient quantity to complete all related experiments. For each sample, vacuum-dried samples were dissolved in injection solvent containing eight or more injection standards at fixed concentrations, depending on the platform. The internal standards were used both to assure injection and chromatographic consistency. Instruments were tuned and calibrated for mass resolution and mass accuracy daily.

The UPLC-MS/MS platform utilized a Waters Acquity UPLC and a ThermoFisher LTQ mass spectrometer, which included an electrospray ionization source and a linear ion-trap mass analyzer. The instrumentation was set to monitor for positive ions in acidic extracts or negative ions in basic extracts through independent injections. The instrument was set to scan 99–1000 m/z and alternated between MS and MS/MS scans. The scan speed was approximately six scans per second (three MS and three MS/MS scans). MS/MS scans were collected using dynamic exclusion, a process in which after an MS/MS scan of a specific m/z has been obtained, then that m/z is placed on a temporary MS/MS exclude list for a user-set period of time to allow greater MS/MS coverage of ions present in the MS scan because the instrument will not trigger an MS/MS scan of the same ion repeatedly. Extracts were loaded onto columns (Waters UPLC BEH C18-2.1×100 mm, 1.7 µm) and gradient-eluted with water and 95% methanol containing 0.1% formic acid (acidic extracts) or 6.5 mM ammonium bicarbonate (basic extracts). Columns were washed and reconditioned after every injection.

The samples destined for analysis by GC-MS were dried under vacuum desiccation for a minimum of 18 hours prior to being derivatized under dried nitrogen using bistrimethyl-silyltrifluoroacetamide. Derivatized samples were separated on a 5% phenyldimethyl silicone column with helium as carrier gas and a temperature ramp from 60° to 340°C within a 17-min period. All samples were analyzed on a Thermo-Finnigan Trace DSQ MS operated at unit mass resolving power with electron impact ionization and a 50–750 atomic mass unit scan range.

*Compound Identification, Quantification, and Data Curation*

Metabolites were identified by automated comparison of the ion features in the experimental samples to a reference library of chemical standard entries that included retention time, molecular weight (m/z), preferred adducts, and in-source fragments as well as associated MS spectra and curated by visual inspection for quality control using software developed at Metabolon [4]. Identification of known chemical entities is based on comparison to metabolomic library entries of purified standards. Over 4,000 commercially available purified standard compounds have been acquired and registered into LIMS for distribution to both the LC/MS and GC/MS platforms for determination of their detectable characteristics. Peaks were quantified using area under the curve.

**Supplemental References**

1. English JG, Shellhammer JP, Malahe M, McCarter PC, Elston TC, Dohlman HG. MAPK feedback encodes a switch and timer for tunable stress adaptation in yeast. Sci Signal. 2015;8(359):ra5.

2. Evans AM, DeHaven CD, Barrett T, Mitchell M, Milgram E. Integrated, nontargeted ultrahigh performance liquid chromatography/electrospray ionization tandem mass spectrometry platform for the identification and relative quantification of the small-molecule complement of biological systems. Analytical chemistry. 2009;81(16):6656-67.

3. Sha W, da Costa KA, Fischer LM, Milburn MV, Lawton KA, Berger A, et al. Metabolomic profiling can predict which humans will develop liver dysfunction when deprived of dietary choline. FASEB journal : official publication of the Federation of American Societies for Experimental Biology. 2010;24(8):2962-75.

4. Dehaven CD, Evans AM, Dai H, Lawton KA. Organization of GC/MS and LC/MS metabolomics data into chemical libraries. J Cheminform. 2010;2(1):9.
